# In-Field Performance Evaluation of an IoT Monitoring System for Fine Particulate Matter in Livestock Buildings

**DOI:** 10.3390/s25164987

**Published:** 2025-08-12

**Authors:** Provvidenza Rita D’Urso, Alice Finocchiaro, Grazia Cinardi, Claudia Arcidiacono

**Affiliations:** Department of Agriculture, Food and Environment, University of Catania, Via Santa Sofia n. 100, 95123 Catania, Italy; alice.finocchiaro@unict.it (A.F.); carcidi@unict.it (C.A.)

**Keywords:** low-cost device, environmental monitoring, air quality, particulate matter, dairy barn

## Abstract

**Highlights:**

**What are the main findings?**

- The low-cost sensor has lower reliability compared to the reference device;
- A linear regression model has been developed to correct PM2.5 measurements by the reference device.

**What is the implication of the main findings?**

- Using low-cost multi-parameter sensors represents an affordable solution to monitor PM2.5 in dairy barns;
- IoT tools integrated with air pollutants sensors play a key role in air quality monitoring and potential mitigation strategies.

**Abstract:**

The livestock sector significantly contributes to atmospheric emissions of various pollutants, such as ammonia (NH_3_) and particulate matter of diameter under 2.5 µm (PM2.5) from activity and barn management. The objective of this study was to evaluate the reliability of low-cost sensors integrated with an IoT system for monitoring PM2.5 concentrations in a dairy barn. To this end, data acquired by a PM2.5 measurement device has been validated by using a high-precision one. Results demonstrated that the performances of low-cost sensors were highly correlated with temperature and humidity parameters recorded in its own IoT platform. Therefore, a parameter-based adjustment methodology is proposed. As a result of the statistical assessments conducted on this data, it has been demonstrated that the analysed sensor, when corrected using the proposed correction model, is an effective device for the purpose of monitoring the mean daily levels of PM2.5 within the barn. Although the model was developed and validated by using data collected from a dairy barn, the proposed methodology can be applied to these sensors in similar environments. Implementing reliable and affordable monitoring systems for key pollutants is crucial to enable effective mitigation strategies. Due to their low cost, ease of transport, and straightforward installation, these sensors can be used in multiple locations within a barn or moved between different barns for flexible and widespread air quality monitoring applications in livestock barns.

## 1. Introduction

The livestock sector is a significant source of atmospheric emissions, including ammonia (NH_3_), methane (CH_4_), carbon dioxide (CO_2_), and particulate matter (PM). These emissions have serious implications for animal and human health, as well as contributing to climate change [1]. In livestock barns, PM2.5 is produced from both primary and secondary sources. Primary PM2.5 is directly emitted from animal activity, manure removal, feeding management, and bedding materials [2]. Secondary PM2.5 originates from NH_3_ produced from urine and manure, which reacts with atmospheric acidic compounds to form secondary inorganic aerosols (i.e., ammonium sulphate and ammonium nitrate) [3,4]. In addition, bioaerosols (i.e., bacteria, endotoxins, and fungal spores) have been associated with PM2.5, representing a significant component of airborne matter in confined animal housing [5]. The study of PM2.5 in livestock barns is significant as it represents a key indicator of air quality, and elevated concentrations in livestock housing can negatively affect both animal health and welfare. Several studies [6,7,8] have highlighted the harmful effects of PM2.5 on the respiratory health of agricultural workers and farm animals. Prolonged exposure to high levels of PM2.5 in livestock environments has been associated with an increased prevalence of respiratory such as chronic obstructive pulmonary disease and lung cancer [9].

Reducing these emissions is therefore essential to ensure environmental sustainability and improve indoor air quality, with positive effects on animal welfare, productivity, and occupational health for farm workers. In this context, it is crucial to implement monitoring systems that can detect concentrations of key pollutants in a timely and accurate manner, enabling the adoption of effective corrective measures [10]. Precision Livestock Farming (PLF) is an effective strategy for reducing environmental impacts from the livestock sector. This innovative approach optimises production efficiency by managing resources more rationally, continuously monitoring animal welfare, and systematically collecting data to support more sustainable management decisions [11].

Using sensors and digital technologies enables real-time monitoring of animal health, facilitating early intervention in the event of anomalies and promoting the overall welfare of the herd. These tools also enable the more efficient use of resources such as nutrients, water, and energy, and increase production yields, thereby contributing to the environmental and economic sustainability of livestock farming [12].

Among the technologies, sensors for real-time monitoring of atmospheric gas concentrations are of particular interest. Generally, reference devices are very expensive, which is impractical to make multiple measurements (i.e., in one barn or in more barns). These instruments used in livestock research, such as photoacoustic analysers or analysers based on Fourier transform infrared (FTIR) systems, are designed for high accuracy, precision, and reliability, but their cost and logistical limitations (e.g., size, regular maintenance, and calibration requirements) have reduced their widespread use in routine on-farm monitoring. Consequently, having high-quality data requires large economic investments although the spatial density of measurements may be very limited [13,14].

Therefore, scientific research is actively focused on identifying solutions that achieve an effective balance between reliability, economic sustainability, and ease of use in order to adequately meet application requirements and expected results [15]. In detail, livestock farms’ modernization needs a wide application of industrial sensors that meet the requirements for monitoring air concentrations within a livestock context [16].

Based on the literature [13,17,18] low-cost PM sensors have been analysed and tested across indoor and outdoor applications or under different environmental conditions (i.e., temperature and relative humidity (RH)). While these studies have primarily focused on urban air quality monitoring or, in general, indoor environments, limited research has analysed the application and validation of low-cost PM2.5 measuring devices within livestock housing, where microclimatic conditions, airborne bioaerosols, and PM sources are different compared to urban areas. A limited number of studies [14,19,20,21] have recently explored the use of low-cost sensors in livestock environments. For instance, the study of Kamp et al. [22] investigated a fluorescence sensor for NH_3_ that demonstrated a strong correlation with control data. This makes it a viable option for monitoring barn air quality. Passive samplers, such as the Ogawa passive sampler and the Passive Flux Sampler, have also demonstrated acceptable accuracy in naturally ventilated barns. These alternatives represent valid, cost-effective solutions for assessing air quality in and around dairy barns [16]. In addition, to enable real-time monitoring of environmental parameters (i.e., ammonia, hydrogen sulphide, and PM concentrations), open-source multisensor platforms have been implemented in dairy barns [14], capable of simultaneously tracking various environmental parameters within breeding environments. In this context, Provolo et al. [20] developed and primarily assessed an Internet of Things (IoT) framework based on low-cost sensors to analyse the spatial and temporal distribution of environmental variables in livestock environments (i.e., microclimatic variables, gas sensors, volatile organic compounds). After assessing the device’s performances at laboratory scale by using an accurate device, the IoT system has been applied in three livestock housing conditions (i.e., pigs, dairy cows, and rabbits). However, this study did not carry out direct comparison of the IoT system with reference instruments under in-field conditions. Validating the measurement systems developed is crucial for ensuring the reliability and accessibility of the devices used for experimental research and environmental monitoring in agriculture. This process guarantees the accuracy and consistency of the data collected, enabling farmers to make informed decisions about crop and natural resource management. For example, Nagahage et al. [23] conducted a study in which they employed a low-cost capacitive sensor to automatically acquire soil moisture data. They compared the sensor’s measurements with those obtained through the gravimetric method and a reference sensor. The results demonstrated that such devices can provide reliable data when properly corrected and validated, making them useful tools for precision irrigation. In addition, validating environmental monitoring systems is crucial for ensuring quality and safety in agricultural production. Barbaresi et al. [24] proposed a measurement validation method based on comparing data collected by different sensors with data provided by certified instruments. This allows the reliability of the devices to be assessed, supporting more informed technological choices for developing climate-smart agriculture. Ellis et al. [25] emphasised the importance of incorporating empirical data directly from farms in order to test and refine predictive models. This ensures that the models are representative of real-world conditions, thereby enhancing the effectiveness of sustainable agricultural practices. In summary, validating measurement systems is a vital step for ensuring that the adopted agricultural tools are reliable, affordable, and scientifically valid, thereby significantly contributing to research advancement and the sustainability of agronomic practices.

Therefore, based on the literature, it can be stated that low-cost devices can be precise and accurate under specific conditions; however, their long-term reliability is often limited due to sensor degradation, environmental interferences (especially in livestock barns), or lack of robust correction protocols [21,26].

In the context of livestock farming, there is a critical need for affordable and reliable monitoring systems for both farmers and researchers. In fact, the economic sustainability of barn management often precludes farmers from investing in high-cost air quality monitoring instruments, despite the importance of maintaining optimal environmental conditions for animal health and welfare. With regards to researchers, obtaining representative emission factors and evaluating mitigation strategies requires many measurement devices to carry out measurements at multiple points within a barn and across different barns, preferably in various climatic regions.

Currently, emission factors often derive from studies carried out in Northern Europe (e.g., the Dataman database reported in the study of Hassouna et al., 2022 [27]), whereas local in-field data is essential for accurate inventories and policy development in other regions such as the Mediterranean area.

In this context, the analysis of low-cost sensors for PM2.5 monitoring in barns has not been investigated in depth. This study aims to fill this gap, evaluating the reliability of low-cost sensors for monitoring PM2.5 concentrations in a dairy house. To support the experiment, an IoT system, previously developed and tested in various agricultural contexts involving pigs, cattle, and rabbits [20], was compared to a high-precision device to verify its effectiveness and scalability. In detail, the main objectives are: (i) comparing PM2.5 concentrations acquired by the reference and the low-cost devices; (ii) optimising the performance of the low-cost device though correction equations; (iii) determining PM2.5 concentrations after correction against reference PM2.5 concentrations; and (iv) identifying the potential use in a dairy barn.

## 2. Materials and Methods

### 2.1. Low-Cost Device

**Figure 1:**
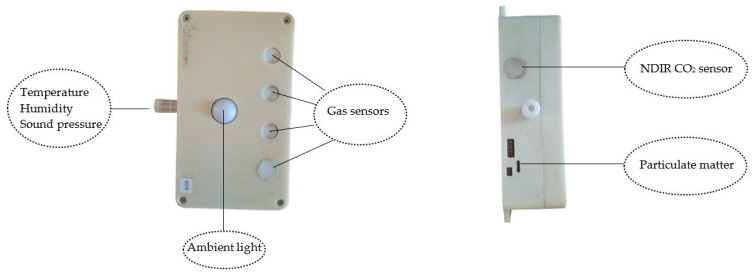
Low-cost sensor device assessed in this study.

The environmental monitoring system used in this experiment, Air Quality N11, was based on multisensor nodes developed by IBT Systems (Milan, Italy) (Figure 1). 

These devices can provide a detailed monitoring of microclimatic conditions and air quality in livestock facilities. Each node is based on a modular, integrated architecture. At the heart of the device is a main board that houses all the electronic components and includes 128 MB of flash memory for storing collected data locally, which is useful in cases of connection interruption. The system is powered by an internal battery which provides 20–25 days of autonomy. The multi-sensor nodes are protected by a cover plate.

Data management and processing are handled by a low-power microcontroller with internal memory and two radio modules. The first module operates via Bluetooth Low Energy (BLE) and is used for configuration and local diagnostics via a mobile app. The second transmits data over long distances at a frequency of 868 MHz to a central gateway. This gateway collects data from multiple nodes and forwards it to a cloud platform, where the information is organised and displayed on analysis dashboards. The nodes are equipped with integrated sensors that can measure various environmental parameters, such as air temperature, air RH, PM, CO_2_, NH_3_, hydrogen sulphide (H_2_S), sound pressure, and volatile organic compounds (VOCs). The technologies used by sensors vary depending on the parameter being measured. For instance, PM and CO_2_ sensors are based on digital technologies such as laser scattering and Non-Dispersive Infrared (NDIR), whereas NH_3_ and H_2_S sensors operate according to electrochemical principles.

Data is acquired at different frequencies according to the parameter: every 10 s for gases (such as NH_3_, H_2_S, and CO_2_), every minute for temperature and RH, and every 15 min for PM and VOCs. The collected data is then averaged over ten-minute time windows and sent to the gateway in the form of compact packets. Further details regarding the low-cost device can be found in the study by Provolo et al. [20]. In the present text, we focus specifically on the measurements collected during the experiment. The parameters monitored were PM2.5 concentration, air temperature, and relative humidity (RH). The sensors integrated into the IBT nodes used to monitor these parameters have the characteristics described in Table 1.

Provolo et al. [20] also provided the average error and standard deviation values for these three parameters, in laboratory conditions. In particular:

- For air temperature, the mean absolute error is 0.04 °C, with a mean relative error of 0.21%. The mean standard deviation is 0.05 °C in absolute terms, and 0.27% in relative terms;
- For RH, the mean absolute error is 0.12%, with a relative error of 0.17%. The absolute standard deviation is 0.15%, while the relative standard deviation is 0.22%;
- For PM2.5, the mean absolute error was 0.31 µg/m^3^, with a mean relative error of 2.46%; the mean standard deviation was 0.41 µg/m^3^, with a mean relative standard deviation of 3.28%.

### 2.2. Reference Device for Fine Particulate Matter (PM 2.5) Measurements

The reference device MP101M (ENVEA, France) was applied to monitor PM2.5 concentrations in the barn. The MP101M is a high-precision instrument designed for the automatic measurement of suspended PM in ambient air. It allows the monitoring of PM10 and PM2.5 concentrations according to the ISO 10473 method, with a measurement range from 0 to 10,000 μg/m^3^. In detail, the PM2.5 sensor features are reported in Table 1. Data was acquired with a sampling frequency of one hour. The overall dimensions of the reference device are 1.06 × 1.20 × 1.32 m, and the reference device inlet is located at 2.70 m height. The measurement principle is based on the attenuation of beta radiation, a highly effective technique that allows the density of airborne particles to be determined independently of their chemical composition. A known volume of air containing PM is drawn in and deposited on a glass fibre filter. An automatic sequential sampling system ensures continuous particle collection, providing high temporal resolution and the ability to monitor changes in concentrations over time. During analysis, a source of beta radiation emits particles that pass through the particle-laden filter. The absorption of this radiation is measured using a Geiger–Müller counter and the data obtained is compared with that of an empty reference filter. As absorption follows an exponential law, this method provides highly reliable and accurate measurements, even in the presence of particles of varying chemical composition.

### 2.3. On-Farm Experiment Description

The experiment was conducted in a loose housing barn with 60 dairy cows located in Ragusa province (southern Italy).

**Figure 2:**
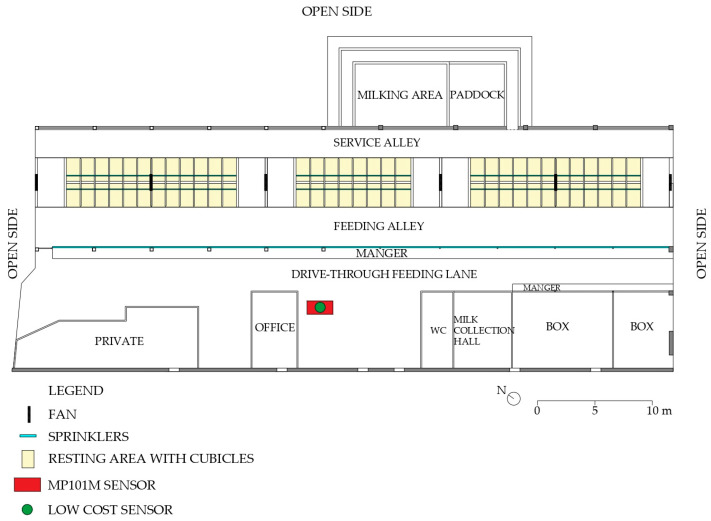
Plan view of the barn and position of fans and sensors. (Source: drawn by the authors.).

The barn had a rectangular floor plan with dimensions of 55.5 m by 21.4 m and it was characterised by three open sides and one side closed by a continuous wall with four openings. The floor was made of concrete and the roof comprised corrugated fibre-reinforced concrete panels. The height of the barn was 4 m at the eaves and 7 m at the ridge. As shown in the floor plan layout (Figure 2), the main functional areas in the barn were the manger, the feeding alley, service alleys, and the resting area. This latter included 64 ‘head-to-head’ cubicles with sand bedding, separated by kerbs and organised into three pens. The milking area with paddock is close to the north-eastern side of the building, while the building also accommodates a milk collection hall, two young-calf boxes, and the farmer’s private facilities (i.e., WC, office, and private areas).

Floor cleaning operations took place daily at around 07:00, while the unifeed ration was distributed once a day at around 10:00. Milking was automated thanks to a robot positioned on the north side of the facility. Ventilation was provided by a combined system of sprinklers and fans in the feeding lane, as well as a misting system with fans in the resting area. In both cases, the fans were inclined at an angle of 20° to the horizontal. Specifically, they were activated at three main times of day: during unifeed distribution at 10:30, in the early afternoon at 13:30, and in the late afternoon at 16:30.

### 2.4. Data Acquisition and Processing

**Figure 3:**
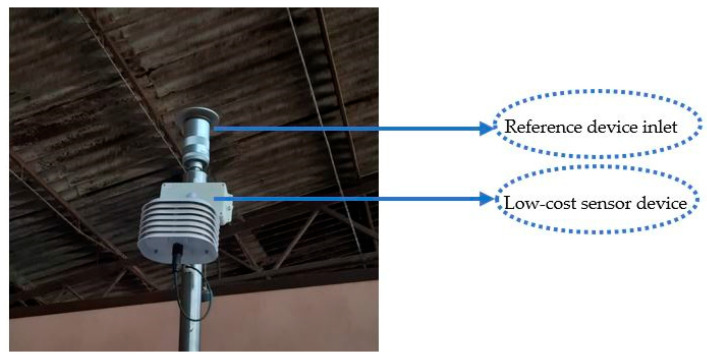
Combination of the low-cost instrument with the reference instrument.

Measurements were taken over a period of 46 days, from 14 March 2025 to 9 May 2025. The measurement devices were located in the centre of the barn (Figure 2 and Figure 3), with the low-cost device position on the measuring filter of the MP101P (Figure 3) at a 2.70 m height. The installation height of the low-cost sensor was chosen to match the inlet height of the reference device to ensure comparability of measurements at the same air sampling point and avoid systematic errors. The high-precision Envea reference instrument operates with a continuous volumetric flow rate. While the Envea draws air through its system by using a pump, this localised suction is negligible in comparison to the natural movement and mixing of ambient air in the open environment where measurements were carried out. Therefore, the flow rate of the reference instrument does not create a significant localised depletion zone of particles that would affect the representativeness of the nearby low-cost sensor’s measurements. Both instruments are sampling from the same well-mixed atmospheric volume, ensuring that the air analysed by both instruments is fundamentally the same ambient air.

PM2.5 concentrations and air temperature and RH were acquired by the reference and low-cost devices. Then, the data was organised in a datasheet, and the mean values of measurements acquired from the low-cost device were compared to the hourly measurements acquired by the reference device.

Data analysis was carried out on climate and PM data acquired by the two devices. In detail, correlation analyses were carried out between PM2.5 concentrations acquired by the two different devices. To improve the reliability of the measurement, a linear regression was carried out to obtain PM2.5 concentrations of the low-cost device adjusted by using the PM values measured by the reference device, and the environmental parameters (i.e., air temperature and relative humidity) acquired by the low-cost device. In addition, error analysis was carried out to assess the reliability of the low-cost device measurements by using data acquired by the reference device as benchmark. A one-way analysis of variance (ANOVA) was applied to assess significant differences between groups of variables (i.e., PM2.5, indoor air temperature, error) acquired with low-cost and reference devices. Then, a post hoc analysis was carried out for each ANOVA, and the mean values were separated by Tukey’s honestly significant difference at *p* < 0.05.

## 3. Results

This section is organised into three subsections, describing the outcomes related to the different PM2.5 concentrations measured by the reference and low-cost devices (Section 3.1), the correction equations to align the PM2.5 concentrations values of the low-cost device to the reference values (Section 3.2), and the statistical-based assessment of the PM2.5 concentrations between the reference values and the adjusted values of the low-cost device (Section 3.3).

### 3.1. Statistical Analyses of Low-Cost Device PM2.5 Concentrations and Temperature Measurements

**Figure 4:**
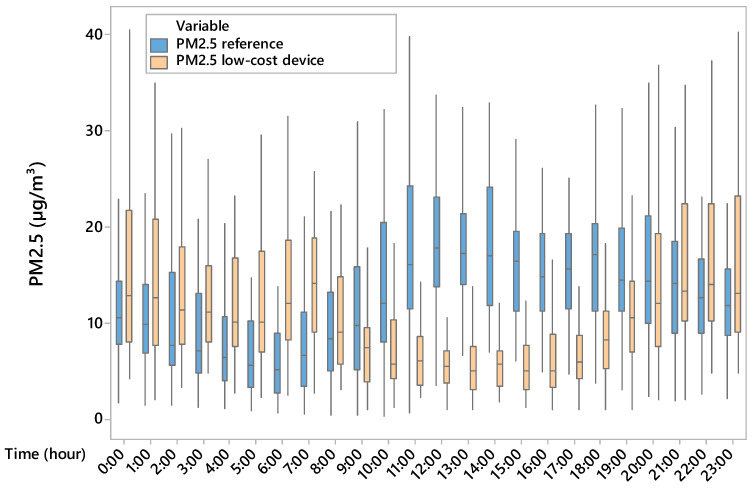
Boxplot of hourly PM2.5 concentrations measured by using the reference (blue) and low-cost devices (pink).

Based on one-way ANOVA, there was a significant difference (*p* < 0.001) between PM2.5 concentrations measured by using the reference device and those acquired by using the low-cost device. In detail, the mean values of PM2.5 were 13.62 µg/m^3^ and 11.61 µg/m^3^ for the reference and low-cost device, respectively. The results showed that the low-cost device underestimated the PM2.5 concentrations with mean values of absolute and relative errors equal to 2 µg/m^3^ and 30%, respectively. Based on the boxplot (Figure 4), PM2.5 concentrations were overestimated during the night and underestimated during the day by the low-cost device; in addition, hourly data boxplot variability of low-cost device measurements was lower during the central hours of the day and overestimated during the night compared to data variability of the reference. Since the boxplot height represents the interquartile range (difference between the third and the first quartiles) the dispersion of half the data is different for the two devices. The low-cost device value underestimation and overestimation produced also a different daily trend of PM2.5 compared to the reference one, which showed many peaks during the day. With regard to indoor air temperature, it was found that temperatures of (18.56 ± 4.93) °C and (17.30 ± 5.83) °C were recorded by the reference and low-cost device, respectively. Although there is a significant difference (*p* < 0.001) between indoor air temperature measured by the reference device and that measured by the low-cost device, the low-cost sensor for air temperature is capable of representing the daily indoor temperature.

**Figure 5:**
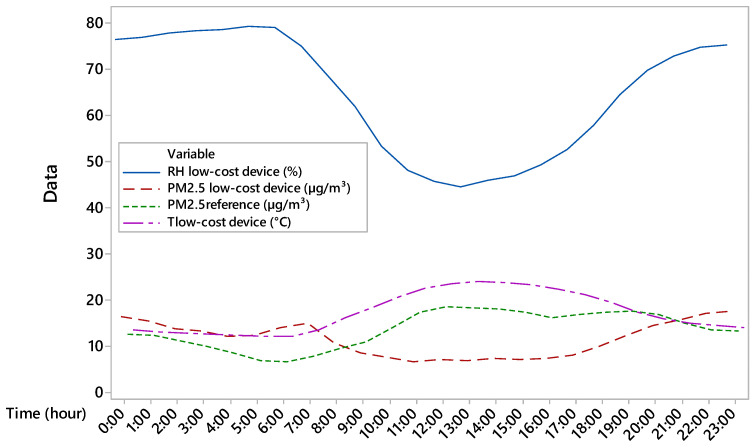
Overlapping between PM2.5 concentrations measured by using reference and low-cost devices and environmental parameters acquired by the low-cost device.

In Figure 5, PM2.5 concentrations acquired by low-cost and reference devices have been overlapped to environmental parameters measured by the low-cost device in order to assess the influence of specific parameters on the PM2.5 measurements. The results show that the PM2.5 concentrations acquired by the low-cost device are influenced by air temperature and RH. Indeed, when the curve of RH decreases, the PM2.5 concentrations detected by low-cost device follow the trend of RH underestimating the reference measure of PM2.5 concentrations. Conversely, when RH increases, PM2.5 concentrations are overestimated. The threshold for minimising the absolute error between reference- and low-cost-based PM2.5 concentrations occurred for a RH value of about 75% and air temperature of about 14.5 °C. When RH was below the threshold of 75% and air temperature exceeded the threshold of 14.5 °C, PM2.5 concentrations were underestimated by the low-cost device, and vice versa. Air temperature contributes to increase the difference between the two PM2.5 trends. The considered threshold is then based on an observed correlation between the accuracy of the low-sensitivity device and air parameters.

### 3.2. Correction Equations for PM2.5 Acquired by the Low-Cost Device

By using the threshold of 75% RH (and 14.5 °C for air temperature), the dataset has been split into two sub-datasets, named D1 and D2 hereafter, by collecting the PM2.5 values for RH ≤ 75% and for RH > 75%, respectively. For each sub-dataset D1 and D2, 70% and 30% of them have been randomly selected to define two sub-datasets for training and testing, named D1_train_, D1_test_, D2_train_, and D2_test_, respectively. These test sets contain data that the models have never seen during their training phase. This separation into train and test sets is fundamental to evaluate the generalisation capability of our correction functions. Indeed, evaluating the model based on the training dataset would yield artificially high accuracy estimates due to potential overfitting. By evaluating on D#test, it has been measured how well the correction function performs on new data under similar environmental conditions.

A regression analysis provided the equations to correct PM2.5 concentrations acquired by the low-cost device:

For RH ≤ 75 (sub-datasets D1_train_), the regression analysis coefficient R^2^(adj) equalled 42.68% and the equation was the following:

PM2.5_ref_ (µg/m^3^) = 0.29 + 0.6888 PM2.5_low-cost_ (µg/m^3^) − 0.0528 RH_low-cost_ (%) + 0.609 T_low-cost_ (°C)

By using this equation, PM2.5 concentrations measured by the low-cost device have been adjusted and the correlation index between PM2.5 values measured by the reference and those by the adjusted low-cost device was 0.656 and 0.783 for the training and testing sub-datasets D1_train_ and D1_test_, respectively.

For RH > 75 (sub-datasets D2_train_), the regression analysis coefficient R^2^(adj) equalled 50.17% and the equation was the following:

PM2.5_ref_ (µg/m^3^) = 4.41 + 0.3774 PM2.5_low-cost_ (µg/m^3^) − 0.1367 RH_low-cost_ (%) + 0.8580 T_low-cost_ (°C)

Using this equation, the PM2.5 concentrations measured by the low-cost device were adjusted in the D2_train_ sub-dataset, resulting in a correlation index of 0.712 with the reference measurements. In the testing sub-dataset D2_test_, the adjusted PM2.5 concentrations showed a slightly lower correlation index of 0.658 compared to the training sub-dataset. This result indicates that the correction equation maintains a good predictive performance when applied to new data, although with a slight decrease in accuracy compared to the training data. Regression analyses, in fact, were designed to correlate the value measured by the less accurate instrument with the more accurate instrument and were not intended to analyse the daily trend of PM2.5, but to test the behaviour of the less accurate instrument in different environmental conditions that may cause misfunctioning.

**Figure 6:**
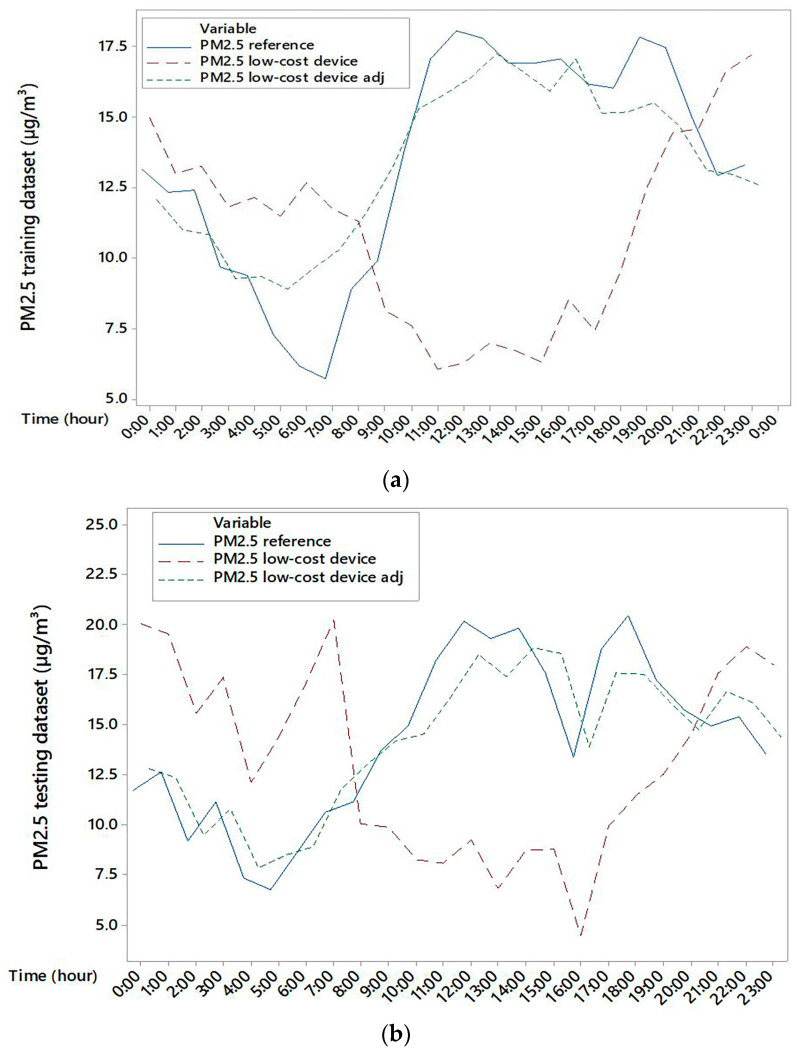
Daily trend of PM2.5 concentrations for reference device, low-cost device, and adjusted low-cost device for training (**a**) and testing (**b**) datasets.

Based on these corrections of PM2.5 concentrations for datasets D1 and D2 with RH values lower and higher than 75%, the correlation indices between PM2.5 concentration of the reference device and adjusted PM2.5 concentrations of the low-cost device were 0.731 and 0.790 for training and testing, respectively. The training dataset (Figure 6a) and testing dataset (Figure 6b) for each hour of the days were represented in Figure 6.

### 3.3. Analysis of Adjusted PM2.5 Concentrations 

**Figure 7:**
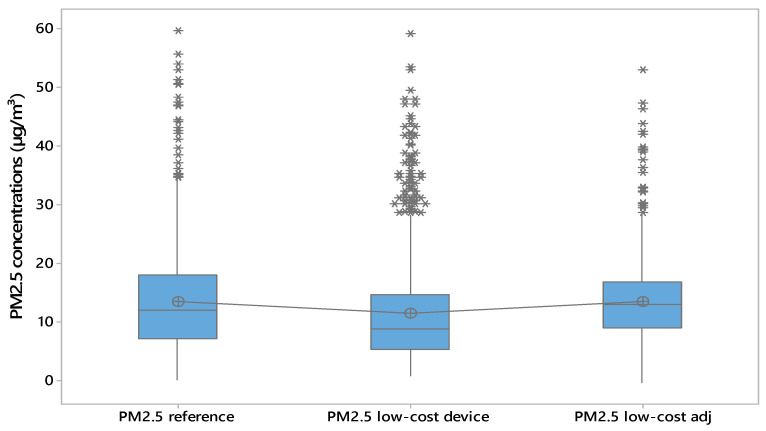
Boxplot of PM2.5 concentrations for the reference device, low-cost device, and adjusted low-cost device.

**Figure 8:**
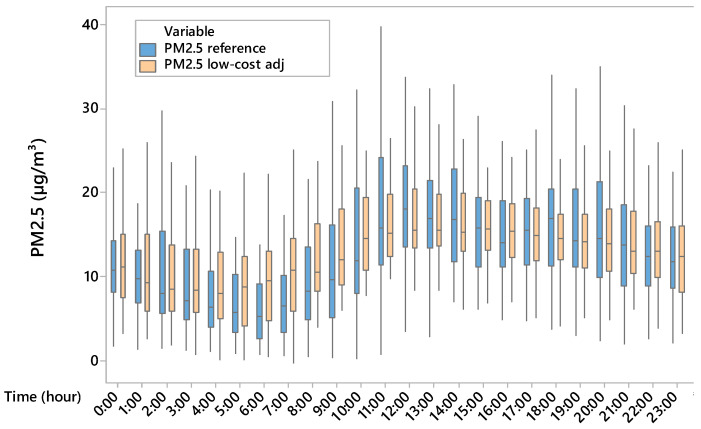
Daily trend of PM2.5 concentrations for reference device (blue) and adjusted low-cost device (pink).

**Figure 9:**
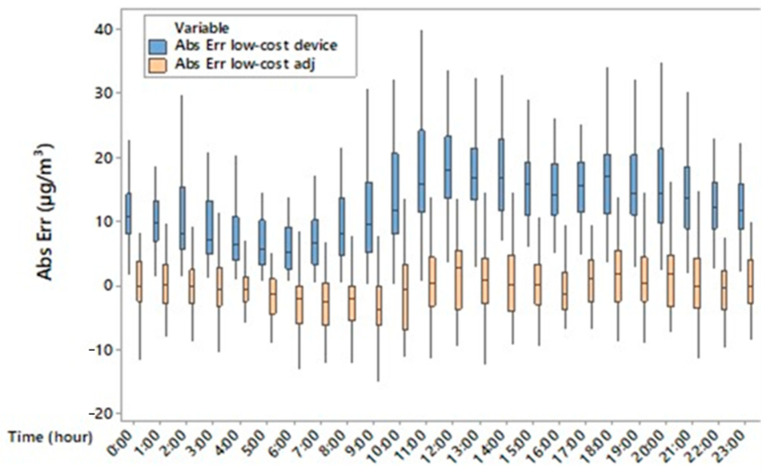
Daily trend of absolute error (Abs Err), calculated as the difference between the PM2.5 concentrations measured by the reference instrument and those measured by the low-cost device (blue), and as the difference between the reference measurements and the adjusted PM2.5 concentrations of the low-cost device (pink).

The correction carried out on the PM2.5 concentrations acquired by the low-cost device provided good results. In fact, the Tukey post hoc test (Table 2) showed no significant difference between the PM2.5 values from the reference device and those from the adjusted low-cost sensor, while differences remained with the unadjusted low-cost data. Based on the boxplot (Figure 7), the median for the unadjusted low-cost device group is not centred within the interquartile range, suggesting a higher degree of skewness in the data distribution compared to the other two groups. In addition, the line connecting the boxplots, which represents the mean PM2.5 concentrations for each group, indicated that the mean values measured by the low-cost device were higher than the corresponding median values shown in each boxplot. This discrepancy between the mean and median in the boxplot of PM2.5 concentrations acquired by low-cost devices suggests a skewed distribution or the presence of outliers in the PM2.5 measurements collected by the low-cost device. Additionally, the daily trends of PM2.5 concentrations for reference device and the adjusted low-cost device (Figure 8) are similar, whereas the absolute error (Figure 9) has a constant trend.

**Figure 10:**
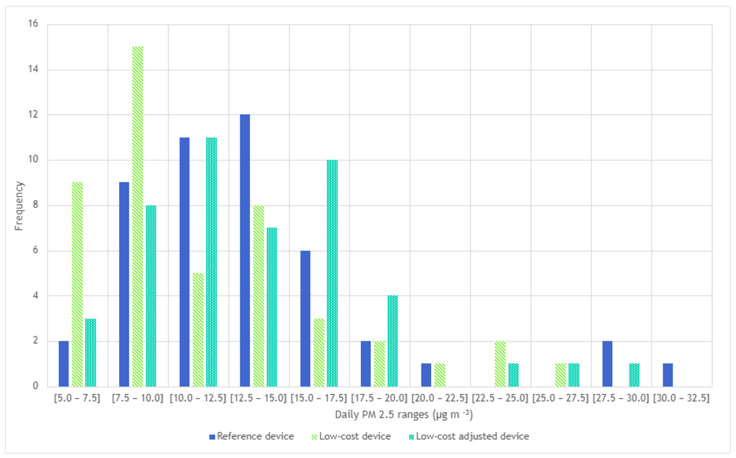
Frequency plot for daily PM2.5 concentrations measured by using reference device, low-cost device, and adjusted low-cost device. The y−axis (‘Frequency’) refers to the actual number of times a daily PM2.5 concentration occurs.

The correction of PM2.5 measured by the low-cost device provided effects on the computation of the daily mean value of PM2.5 concentrations. In fact, Figure 10 shows how 30 µg/m^3^ frequency is not captured by the low-cost device, and the range from 10 to 17.5 µg/m^3^ is highly underestimated by 75%.

## 4. Discussion

The obtained results indicate that, while the considered low-cost sensors have demonstrated a reasonable ability to monitor average PM2.5 levels in livestock environments, correction models based on climatic parameters are required to achieve reliability comparable to reference devices. This challenge is increasingly being addressed by integrated systems based on IoT technologies, which enable real-time environmental monitoring and support predictive, automated decision-making processes. Recent studies, such as that of Abu et al. [28], have highlighted the effectiveness of IoT platforms in continuously collecting environmental and physiological data, thereby facilitating more intelligent and sustainable herd management practices. Similarly, Danev et al. [29] have focused on developing an air quality monitoring system for PLF using low-cost sensors integrated into an IoT platform. These studies have emphasised the importance of in-depth air quality analysis that considers different pollutants, environmental factors, and animal welfare.

### 4.1. Methodological Remarks

Figure 5 of this research study showed how the response of the low-cost sensor was strongly affected by the air RH and T values, with a reversal of behaviour around the 75% and 14.5 °C thresholds, respectively. These were used as thresholds to split the data into D1 and D2 datasets, and to build two separate regression models. Below this value, the sensor tends to underestimate PM2.5, while above it, it overestimates. This is consistent with the findings of Nadali et al. [30] which documented the significant impact of climatic parameters (especially RH) on sensor response in rural settings. They argue that, despite an initial error, low-cost sensors can be valid tools for field applications, particularly when integrated with predictive models that consider local microclimatic conditions. In our case, we developed a differentiated correction model for the two subgroups (RH ≤ 75% and RH > 75%), which increased the correlation coefficient between the corrected and reference data to 0.79 for the test dataset (see Figure 6). This confirms that integrating environmental parameters into correction models can significantly improve the reliability of low-cost sensors in dynamic environments such as dairy barns. Li et al. [31] used low-cost sensors based on laser scattering technology which exhibit significant fluctuations in PM2.5 measurements due to interaction with RH and temperature, particularly in enclosed spaces such as greenhouses or dairy barns. Their study showed that, for RH > 70%, the sensors systematically overestimate the particle concentration, while for lower RH, they tend to underestimate it.

Regarding the random assignment to D#train and D#test sets, the random assignment of data points from D1 into D1train and D1test, and similarly for D2, is a standard practice in machine learning and statistical modelling. Its purpose is to ensure that the test set is representative of the overall variability within its respective humidity and temperature range (D1 or D2). This allows us to evaluate how well the trained correction model generalises to unseen data within that specific range. Indeed, in this study correction functions were developed for a low-sensitivity instrument that inherently behaves differently under distinct environmental conditions (specifically, high vs. low RH, as well as low vs. high air temperature).

By splitting the data based on air RH and temperature thresholds, a change in the physical response of the sensor has been acknowledged. The daily cycle of RH and temperature causes data to fall into these two distinct datasets (D1 and D2). Within each dataset, random sampling for training and testing ensures that the correction model trained on D#train is tested on a subset of data from the same humidity and temperature conditions (D#test) that it has not previously encountered during training. This provides an estimate of the correction model’s performance within each specific RH and temperature range. The approach explicitly deals with two different underlying behaviours of the low-sensitivity sensor under different humidity/temperature conditions.

The two regression analyses performed (for D1 and D2) resulted in two distinct correction functions, each optimised to perform best within its specific humidity and temperature range. Regression analyses are used to test the performance of the less accurate instrument under different environmental conditions that can cause malfunctions. The identified change in behaviour at high humidity (and low temperature) is precisely why two separate models are necessary.

Discontinuity of the two regressions in the threshold can be expected (and is acceptable for this application) because of the sensor’s physics. The low-cost sensor likely exhibits a significant, non-linear change in its response characteristics (e.g., moisture absorption by sensing elements, condensation effects) once a certain humidity/temperature threshold is crossed. This physical change can manifest as an abrupt shift in the relationship between the low-sensitivity reading and the high-precision reading. The best possible correction within each operational range is preferred rather than a mathematically smooth but physically inaccurate transition.

Moreover, when modelling a system that has fundamentally different operating modes or physical characteristics in different regimes, it is often more accurate to develop separate models for each regime rather than forcing a single, continuous model that would perform poorly across the entire range.

### 4.2. Sensor Performance and Correction Models

Figure 3 shows that the hourly variability of PM2.5, which is linked to livestock activities such as feed distribution and fan activation, is not always captured by the low-cost device in terms of hourly analysis. In our study, underestimation during the warmer hours of the day and overestimation at night also suggest a differentiated sensor response depending on thermo-hygrometric conditions (Figure 5 and Figure 6). This is evident from the position of the uncentred median in the boxplot relative to the sensor, suggesting greater asymmetry in the data distribution. In contrast, at cooler times, the larger boxplot indicates greater agreement between values measured by the low-cost device and the reference system. In terms of temporal variability, our hourly measurements (Figure 4) revealed that the low-cost sensor tends to smooth out concentration peaks in the middle of the day. This behaviour is consistent with the observations of Joo et al. [32], who compared several low-cost and reference PM2.5 sensors in indoor environments and found that hourly variability is often underestimated by low-cost devices, especially when RH exceeds 60%. [32] also pointed out that many low-cost sensors exhibit a nonlinear response to abrupt temperature changes. This behaviour was also observed in our dataset, particularly during the morning (7–10 am) and afternoon (2–5 pm), which coincided with fan activations in the barn.

In our study, the low-cost device was unable to accurately track the daily PM2.5 curve due to inconsistent over- and underestimation. However, introducing a correction equation mitigated these weaknesses, significantly improving the instrument’s measurements.

Notably, the correction models were defined in relation to the climate parameters measured by the low-cost device itself, since this allows improving correction effectiveness due to a parameter acquisition closer to the PM2.5 sensor location. This approach is also justified by the fact that, from the perspective of an end user, such as a farmer, the only data available is that obtained from the IoT multi-sensor device. Therefore, it is preferable that the correction model uses data acquired by the same device to provide corrected values to the user without the need for external sensors.

In terms of future research, IoT systems could be equipped with machine learning algorithms to optimise PM2.5 measurements further. The sensor used in this study is based on laser scattering technology; however, other operating principles, such as optical absorption, electro-analytical chemistry, and metal oxide semiconductors, can be found in the literature [16,33,34]. When integrated into IoT systems, such sensors can offer advanced functionalities, such as multi-point monitoring and real-time alarm generation. This makes them a useful tool for both researchers and farmers [20].

In the research field, low-cost devices integrated with correction models and IoT system can be useful to monitor spatial variability at different location in the barn and estimate PM2.5 emission factors on dairy farms. This would contribute to updating emission inventories and evaluating innovative mitigation strategies. Current strategies for reducing PM include controlled mechanical ventilation, water misting, using additives in bedding, and using specific feeds [35]. However, recent approaches also include nature-based solutions, such as green walls, which act as natural biofilters and reduce PM concentrations inside livestock buildings [36].

### 4.3. Practical Implications

From an application perspective, a corrected low-cost device could be used to monitor compliance with environmental regulations and support research into determining PM2.5 emission factors. It is necessary to check compliance with PM2.5 exposure limits directly with farmers. According to the World Health Organization’s [37] guidelines, the recommended daily limit for PM2.5 is 15 µg/m^3^, whereas Directive (EU) 2008/50/EC sets an annual limit of 25 µg/m^3^ at a European level. Figure 10 shows that, according to the reference instrument, PM2.5 concentrations exceeded the threshold of 15 µg/m^3^ on 12 days and the threshold of 25 µg/m^3^ on 3 days. Based on monitoring with the low-cost sensor, six and one exceedance days were identified. In contrast, correcting the PM2.5 values contributed to 12 and one day of exceedances of the 15 and 25 µg/m^3^ limits, respectively. In such a case, farmers could promptly intervene with mitigation strategies to ensure favourable environmental conditions for animal and worker health and enhance the overall environmental sustainability of the agricultural products [38,39,40].

This finding confirms that, once corrected, the measurements from the device can be used to monitor regulatory limits, as predicted by García et al. [41]. In their study, they compared the average PM2.5 concentrations to the 24-h National Ambient Air Quality Standard (NAAQS) value of 35 µg/m^3^. The results showed that eight stables had at least one measurement above this value, and that in half of these, the mean and median values also exceeded the daily NAAQS limit. However, other studies, such as that of D’Urso et al. [42], reported mean errors of low-cost instruments as a function of distance. In the case of NH_3_, they report average errors of 3.4% near the ground and up to 59% at higher altitudes, as well as CO_2_ errors of up to 28%. Janke et al. [21] generally observed errors of less than 7% for greenhouse and environmental gases, with maximum peaks of up to 67%, thus confirming the reliability of low-cost sensors in real agricultural settings.

This research study focused on the measurement of accuracy and precision of low-cost sensors through correction models for PM2.5 measurements, thereby improving their utility while maintaining their advantages of portability, ease of installation, and affordability. In addition, this study contributed to a wider deployment for spatial monitoring within barns, multi-site and multi-barn data collection for robust regional assessments, and practical application by farmers and researchers who require accessible yet scientifically valid monitoring solutions.

## 5. Conclusions

This study evaluated the performance of low-cost sensors for monitoring PM2.5 concentrations in livestock environments, specifically comparing them against a high-precision reference instrument. The primary goal of this study was to provide the most accurate corrected PM2.5 value given the current environmental conditions.

A linear regression model was developed by using data from the low-cost sensor, with adjustments made for RH and temperature readings from the sensor itself. This modification resulted in a substantial enhancement of the sensor’s precision.

The encouraging outcomes of the present study provide a basis for future research investigations utilising low-cost sensors in livestock barns. A key area for future research is to assess the differences in PM2.5 concentrations across various barns by using the same low-cost sensor location setup. This would provide valuable insights into how factors such as barn design, ventilation systems, animal density, and specific management practices influence air quality in different livestock housing conditions. It is crucial to comprehend this variability in order to formulate targeted mitigation strategies. Additionally, the multi-node functionality of these low-cost instruments, which allows for simultaneous measurements at different locations, makes them optimal for the evaluation of the efficacy of air pollutant mitigation strategies. These sensors could be installed before and after implementing interventions, such as improved ventilation, different feed additives, or alternative bedding materials, to quantify their impact on PM2.5 and other pollutant concentrations in real-time. This capability would facilitate data-driven decision-making, thereby optimising barn air quality.

## Figures and Tables

**Table 1:** Sensor features for PM2.5, air temperature, and RH sensors from the low-cost device and PM2.5 from the reference device.

Sensor	Measurement Range	Resolution	Accuracy
PM2.5 _reference device_	0–10,000 μg/m^3^	≤2 µg/m^3^ (24-h averages)	2.5 µg/m^3^
PM2.5 _low-cost device_	0–1000 µg/m^3^	1 µg/m^3^	10 µg/m^3^
Temperature _low-cost device_	−40 to +125 °C	0.01 °C	0.3 °C
RH _low-cost device_	0–100%	0.1%	3%

**Table 2:** PM2.5 concentration values for reference device, low-cost device, and adjusted low-cost device.

Variable	Mean	SE Mean	Minimum	Maximum
PM2.5, reference	13.64 ^a^	0.27	0.27	59.80
PM2.5, low-cost device	11.59 ^b^	0.27	1.00	59.25
PM2.5, low-cost device adj	13.57 ^a^	0.20	0	53.10

* Groups that do not share the same letter (a,b) are significantly different.

## Data Availability

Data will be available on request to the corresponding author.

## Abbreviations

The following abbreviations are used in this manuscript:

PM2.5	Particulate Matter 2.5
PLF	Precision Livestock Farming
NH3	Ammonia
IoT	Internet of Things
RH	Relative humidity
